# Inhalation of ultrafine carbon particles alters heart rate and heart rate variability in people with type 2 diabetes

**DOI:** 10.1186/s12989-014-0031-y

**Published:** 2014-07-16

**Authors:** Rathin Vora, Wojciech Zareba, Mark J Utell, Anthony P Pietropaoli, David Chalupa, Erika L Little, David Oakes, Jan Bausch, Jelani Wiltshire, Mark W Frampton

**Affiliations:** 1 Department of Environmental Medicine, University of Rochester Medical Center, Rochester, NY, USA; 2 Department of Medicine, University of Rochester Medical Center, Rochester, NY, USA; 3 Department of Biostatistics and Computational Biology, University of Rochester Medical Center, Rochester, NY, USA; 4 Pulmonary and Critical Care, University of Rochester Medical Center, 601 Elmwood Ave, Rochester, 14642, NY, USA

**Keywords:** Air pollution, Ultrafine particles, Diabetes, Heart rate, Heart rate variability, Human, Cardiac

## Abstract

**Background:**

Diabetes may confer an increased risk for the cardiovascular health effects of particulate air pollution, but few human clinical studies of air pollution have included people with diabetes. Ultrafine particles (UFP, ≤100 nm in diameter) have been hypothesized to be an important component of particulate air pollution with regard to cardiovascular health effects.

**Methods:**

17 never-smoker subjects 30–60 years of age, with stable type 2 diabetes but otherwise healthy, inhaled either filtered air (0–10 particles/cm^3^) or elemental carbon UFP (~10^7^ particles/cm^3^, ~50 ug/m^3^, count median diameter 32 nm) by mouthpiece, for 2 hours at rest, in a double-blind, randomized, crossover study design. A digital 12-lead electrocardiogram (ECG) was recorded continuously for 48 hours, beginning 1 hour prior to exposure.

**Results:**

Analysis of 5-minute segments of the ECG during quiet rest showed reduced high-frequency heart rate variability with UFP relative to air exposure (p = 0.014), paralleled by non-significant reductions in time-domain heart rate variability parameters. In the analysis of longer durations of the ECG, we found that UFP exposure increased the heart rate relative to air exposure. During the 21- to 45-hour interval after exposure, the average heart rate increased approximately 8 beats per minute with UFP, compared to 5 beats per minute with air (p = 0.045). There were no UFP effects on cardiac rhythm or repolarization.

**Conclusions:**

Inhalation of elemental carbon ultrafine particles alters heart rate and heart rate variability in people with type 2 diabetes. Our findings suggest that effects may occur and persist hours after a single 2-hour exposure.

## Background

Many studies have shown associations between both short- and long-term exposure to air pollution and increased all-cause morbidity and mortality [[1]]. Exposures to fine particles (PM) in ambient air are associated with an increased risk of cardiovascular events [[2]-[5]], and with changes in several cardiovascular indices, including heart rate, heart rate variability (HRV), and blood pressure. However, the precise mechanisms for cardiovascular morbidity secondary to air pollution remain unknown [[6]].

Moreover, little is known about effects in potentially susceptible people such as those with diabetes. People with diabetes may be at increased risk for health effects of particulate air pollution [[7],[8]]. The risk of coronary artery disease in diabetics is 2 to 4 times that of the general population. Diabetics have impaired endothelial function, in part due to a functional nitric oxide (NO) deficiency [[9]].

Ultrafine particles (UFP, ≤100 nm in diameter) have been hypothesized to be an important component of PM with regard to cardiovascular health effects. The physical characteristics of UFP suggest they may carry reactive molecules into the lung, and may translocate to other organs via the blood [[10],[11]]. UFP in the air have a much higher number concentration and surface area than larger particles at the same mass concentration. For example, in order to achieve a low airborne mass concentration of 10 μg/m^3^, 2.4×10^6^ 20 nm UFP/cm^3^ are required compared to 1 particle/cm^3^ of 2.5 μm particles [[12]]. UFP also have a higher fractional pulmonary deposition than fine particles [[13],[14]]. UFP have been shown to cross cell membranes, enter cells, and enter the systemic circulation, although transport beyond the lung has not been definitively shown in humans [[15]]. It has been hypothesized that UFP deliver reactive oxygen species to the vascular endothelium, resulting in oxidative stress, with increased production of pro-inflammatory substances and reduced anti-oxidant capacity, all contributing to endothelial dysfunction and increased atherogenesis [[16]-[18]].

Despite these concerns, there are relatively few data on the health effects of exposure to UFP. A recent expert panel report [[19]] found a relative paucity of epidemiological and clinical data specific for UFP, in part because of limited ambient monitoring, marked spatial variability in UFP concentrations, and technical challenges in performing clinical exposure studies. The report cited a need for additional controlled laboratory exposure studies that target UFPs of known source and chemical composition.

The aim of this study was to determine, in subjects with type 2 diabetes, the effects of inhalation of ultrafine particles consisting of elemental carbon (as surrogates for UFP of combustion origin) on a series of electrocardiogram (ECG) parameters describing changes in HRV; repolarization duration, morphology, and variability; and the ST segment. To date, there have been no clinical studies that have evaluated ECG changes after exposure to UFP in diabetics. We hypothesized that controlled exposure to laboratory generated carbon UFP in people with type 2 diabetes alters cardiac function detectable via continuous ECG. UFP exposure reduced high-frequency heart rate variability, and unexpectedly increased the average heart rate 24 to 48 hours after exposure, when compared with air exposure. We previously described UFP effects on blood measurements of platelet function in these subjects [[16],[20]].

## Results

### Subjects

There were 19 subjects (9 men and 10 women) enrolled in the study and all subjects underwent exposures to both clean air and laboratory generated UFP. The characteristics of these subjects have been described previously [[16]]. The mean ± SD age was 45.9 ± 9.5 years ranging from 31 to 59 years. They had type 2 diabetes diagnosed for 4.4 ± 5.2 years. 12 subjects managed their diabetes with oral agents (glipizide, glyburide, metformin, pioglitazone), 2 with insulin, 2 with insulin and an oral agent, and 3 with diet alone. Eight of the subjects were on medication for hypertension (hydrochlorothiazide, valsartan, lisinopril, ramipril). HbA1c levels ranged from 5.6 to 11.0% with mean ± SD 7.6 ± 0.3%. For technical reasons, ECG data were not available for 2 of the 19 subjects.

### ECG changes during prespecified 5-minute segments

Table 1 shows selected ECG parameters at baseline prior to clean air exposure in the current subjects. Also provided for reference are the same parameters, measured under similar conditions, in healthy, younger, non-diabetic subjects from a prior study in our laboratory. SDNN (standard deviation of normal-to-normal sinus beat intervals), rMSSD (root mean square of successive differences in NN intervals), HF (high frequency power, 0.15–0.40 Hz) and LF (low frequency power 0.04–0.15 Hz) were lower in the older, diabetic subjects, as expected.

**Table 1:** HRV parameters for subjects with diabetes in this study, and for healthy, younger, non-diabetic subjects tested under similar conditions*

	**Current subjects**	**Non-diabetic subjects**
	**N = 17**	**N = 23**
	**Age 45.9 (9.5) yr**	**Age 28.5 (7.5) yr**
NN (ms)	907 (143)	914 (149)
SDNN (ms)	38 (18)	66 (41)
rMSSD (ms)	33 (23)	65 (57)
HF (ms^2^)	555 (913)	2380 (4222)
LF (ms^2^)	436 (533)	1580 (2159)
LF/HF	2.9 (5.5)	2.0 (2.2)
HF (nu)	44 (27)	44 (22)
LF (nu)	43 (22)	49 (21)

*5 min recordings supine at rest prior to air exposure. Non-diabetic data from subjects in our prior studies [[35]]. Means (SD).

**Figure 1:**
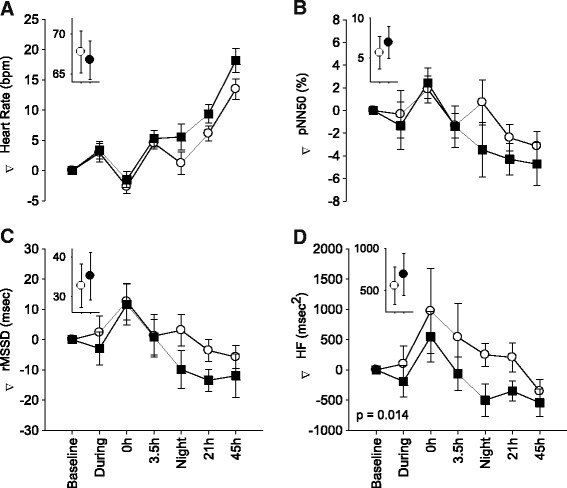
**ECG changes recorded during pre-specified 5 minute segments.** Mean ± SE change from pre-exposure baseline in heart rate **(A)**, pNN50 **(B)**, rMSSD **(C)**, and HF **(D)** in subjects exposed to elemental carbon UFP (solid boxes) and filtered air (open circles), during and at specified time intervals after exposure. The insets show actual baseline values. P-value is for mixed models main effect of exposure.

The heart rate (inverse of the normal-to-normal beat, or NN, interval) increased 21 and 45 hours after exposure to both clean air and UFP, compared with earlier time points (Figure 1A). The increase was slightly but not significantly greater after UFP, beginning during the night after exposure, and persisting at the 21- and 45-hour time-points (mixed models, p = 0.09). There were no differences in heart rate at 0 or 3.5 hours after exposure. The increases in heart rate were associated with decreases in pNN50 (proportion of NNs that differ by more than 50 ms, divided by the total number of NNs) and rMSSD; reductions were greater following UFP than air exposure, but not significantly so (Figure 1B-C). The HF component of the frequency-domain spectrum of HRV decreased significantly with UFP exposure (mixed models, p = 0.014) (Figure 1D), without a significant change in the LF/HF ratio. Other components of the frequency-domain HRV analysis did not show clear differences.

In the 5-minute monitoring segments, there were no statistically significant effects on repolarization duration corrected for heart rate (QTc Bazett), or on QTpeak corrected for heart rate. There were no significant changes in T wave amplitude or T wave complexity, or in the ST segments.

### ECG changes during extended monitoring periods

In the analyses of the extended monitoring periods (0–9, 9–24, 24–48 hours from the beginning of exposure), the 0–9 hour recording interval was used as the baseline for comparison with the subsequent intervals.

**Figure 2:**
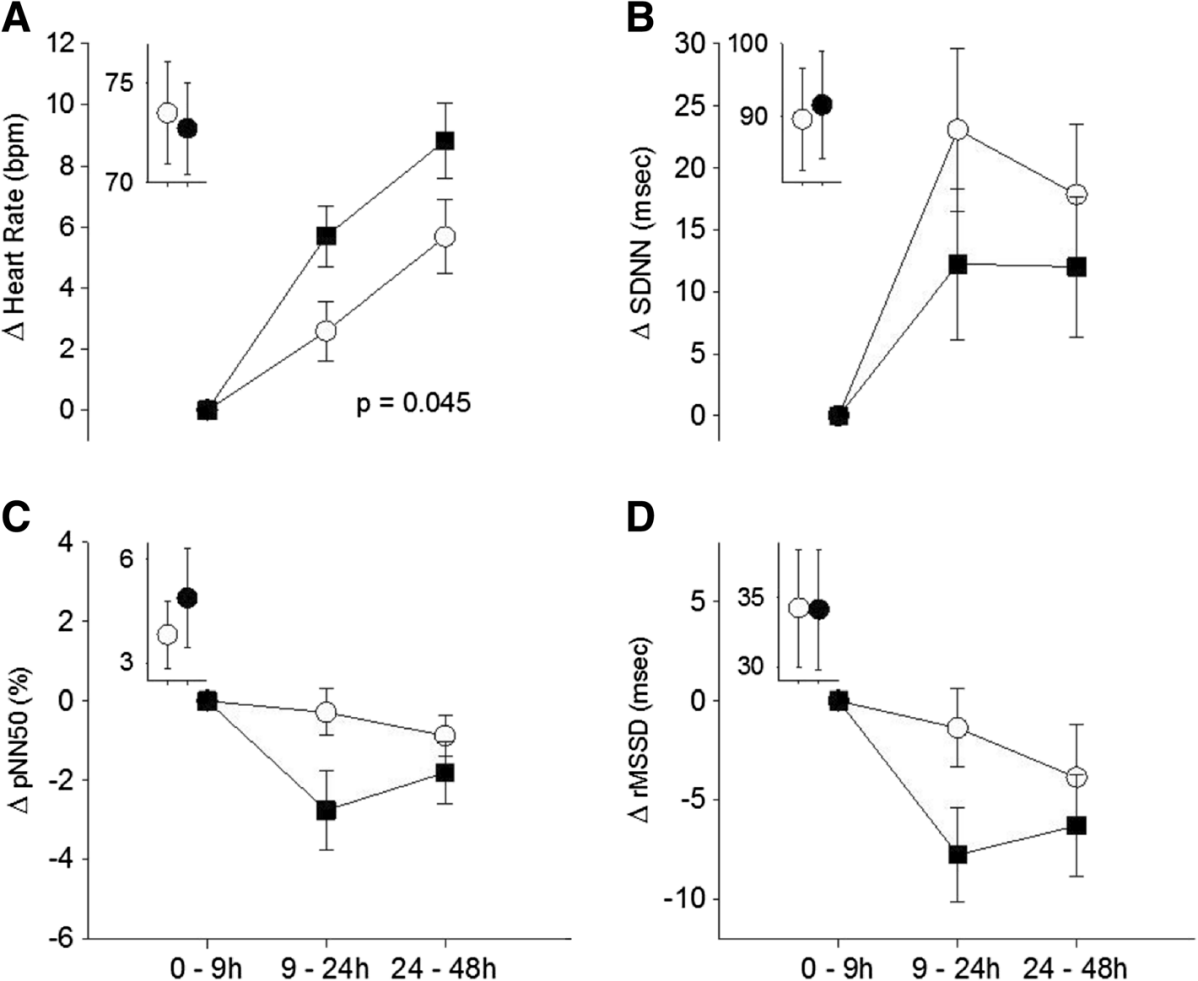
**ECG changes during longer monitoring periods.** Mean ± SE change from first monitoring period (0–9 hours, which included the exposure and first few hours afterward) to subsequent 2 monitoring periods, in heart rate **(A)**, SDNN **(B)**, pNN50 **(C)**, and rMSSD **(D)** in subjects exposed to elemental carbon UFP (solid boxes) and filtered air (open circles). The insets show actual mean values for the 0–9 hours monitoring period, which was used as the baseline. P-value is for mixed models main effect of exposure.

Similar to the findings with the 5-minute segments, UFP exposure increased the heart rate to a greater extent than air exposure (Figure 2A), and the differences were statistically significant (mixed models, p = 0.045). This was paralleled by lower SDNN, pNN50, and rMSSD after UFP exposure, although none were significant (Figure 2B-D). There were no significant changes in QTc interval and T wave amplitude, markers of ventricular repolarization.

**Figure 3:**
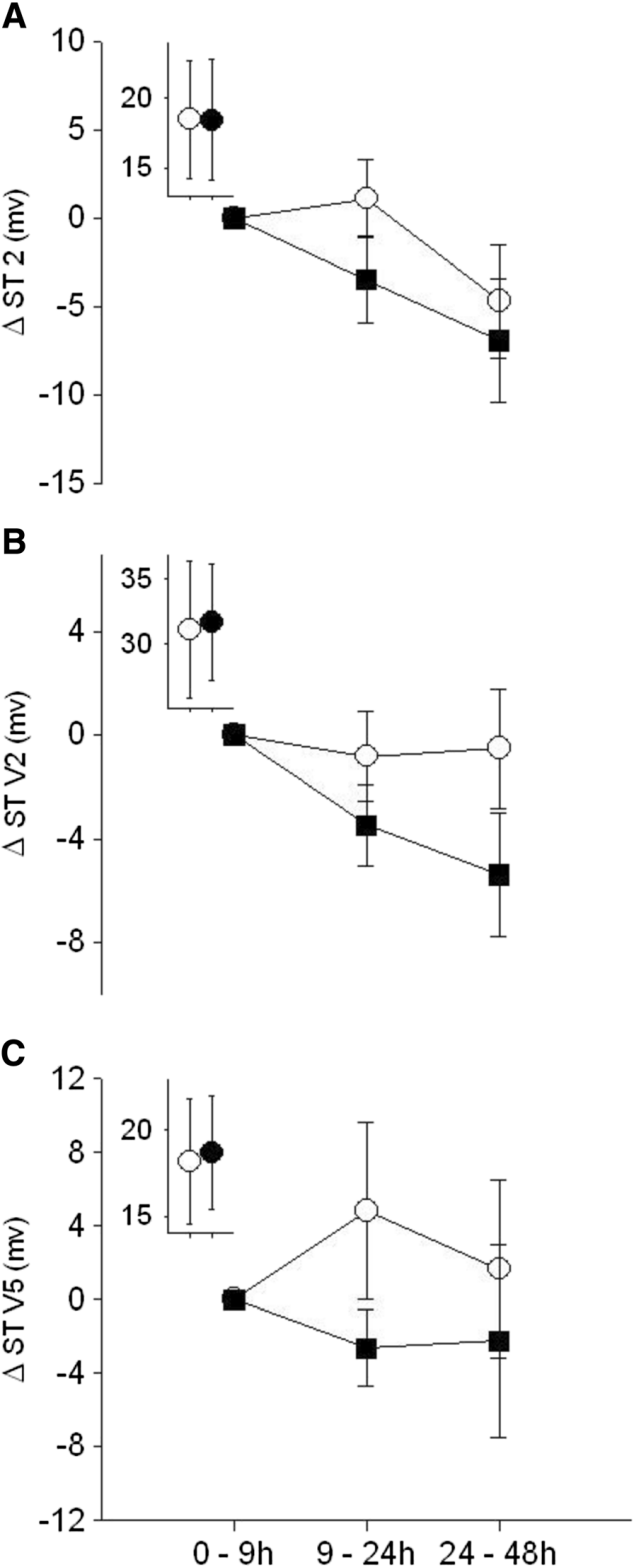
**ECG changes during longer monitoring periods.** Mean ± SE change from first monitoring period (0–9 hours, which included the exposure and first few hours afterward) to subsequent 2 monitoring periods, in the ST segment of the ECG for lead 2 **(A)**, lead V2 **(B)**, and lead V5 **(C)**, in subjects exposed to elemental carbon UFP (solid boxes) and filtered air (open circles). The insets show actual mean values for the 0–9 hours monitoring period.

The long-term recordings also showed small non-significant reductions in the ST segments after UFP exposure, consistently in all three leads (Figure 3). There were a few marginally significant gender and age interactions in the secondary analysis, but overall there was no convincing evidence for consistent differences by age or gender.

Our subjects had very few arrhythmias or premature cardiac beats, as expected because they were required to have a normal baseline ECG. There was no evidence for an increase in arrhythmias following UFP exposure compared with air exposure. Similarly, we found no evidence for effects on heart rate turbulence or deceleration capacity, although the rarity of abnormal beats severely limited our analysis of those parameters.

The blood pressure and heart rate measurements taken with the automated device did not differ significantly between UFP and air exposure, although the heart rate increased about 2 beats per minute more after UPF than after air at the last 2 time points (p = 0.14), similar to the 5-minute ECG data (Figure 1).

## Discussion

Several epidemiological studies suggest that diabetes confers increased risk for health effects from air pollution exposure [[7],[8],[19]]. We previously reported that UFP exposure transiently increased blood platelet activation and levels of circulating von-Willebrand factor [[16]], consistent with effects on vascular endothelium that promote coagulation. Diabetes also causes autonomic dysfunction, associated with reductions in both time and frequency domain variables of HRV [[21]]. Impaired parasympathetic autonomic function reduces vagal tone, which can blunt the high-frequency HRV response. As shown in Table 1, ECG parameters at baseline in our subjects with diabetes differed from healthy, younger, nondiabetic subjects in our prior studies. These findings indicate that cardiac physiology and autonomic control of the heart are altered in people with diabetes, potentially increasing susceptibility to air pollution.

In otherwise healthy people with type 2 diabetes, we found that inhalation of elemental carbon UFP while resting caused a delayed increase in heart rate and a reduction in HF HRV, in comparison with clean air exposure. There were also small non-significant reductions in pNN50, rMSSD, and SDNN associated with UFP exposure. We found no convincing effects on parameters of cardiac repolarization or cardiac rhythm. In the prolonged recordings, there were small ST depressions in all 3 of the precordial leads that were analyzed, but none of the changes were statistically significant. These findings provide further evidence of an effect of UFP inhalation on the autonomic nervous system in people with diabetes, with reductions in vagal relative to sympathetic influences.

The UFP influence on heart rate was delayed in our study, and appears to be an accentuation of the increase in heart rate that occurred as the subjects left the Clinical Research Center and returned to their home environment. As shown in Figures 1A and 2A, the mean heart rate increased 24 and 48 hours after both air and UFP exposure, probably reflecting increased physical activity and stress associated with the subjects’ return home. The increase in heart rate was greater following UFP exposure then air. The decreases in HF HRV (Figure 1D), and the trends toward decreases in the other markers of HRV associated with UFP exposure (Figures 1B,1C, and 2B,2C,2E), were most pronounced during the night following the exposure, when subjects were presumably sleeping. Parasympathetic influences predominate during sleep, slowing the heart rate and increasing heart rate variability [[22]]. In our study, this increase in parasympathetic tone appeared to be blunted following UFP exposure. We postulate that, in diabetics who have abnormal autonomic function, UFP exposure further shifts autonomic balance toward sympathetic and away from parasympathetic influences. The delay in the effects on heart rate suggests this is not a direct effect of particles on nerve endings within the respiratory epithelium, but a response to a cascade of events, the nature of which is incompletely understood at this time.

One could speculate regarding a number of pathways for delayed effects on heart rate. Some of our subjects were obese and could have had sleep apnea. It is possible that the brief UFP exposure altered sleep dynamics or worsened sleep-disordered breathing in the nights immediately following exposure, thereby blunting the normal increase in parasympathetic tone that occurs during sleep [[23]]. Increased systemic inflammation as a result of UFP exposure could alter autonomic tone, although we found no increases in the circulating inflammatory marker C reactive protein in these subjects [[16]]. The small, nonsignificant decreases in the ST segment (Figure 3) could be consistent with an effect on the myocardium, as a result of subtle changes in cardiac perfusion, afterload, or preload. Changes in atrial repolarization could also affect the ST segment, but this is unlikely in the absence of effects on ventricular repolarization (T wave and QTc). The mean increases in heart rate were small (approximately 3 beats per minute) and not clinically important for people without heart disease. However, even small effects on heart rate may be clinically important. Most episodes of cardiac ventricular arrhythmia, or myocardial ischemia, are preceded by an increase in heart rate [[24]]. Increases in heart rate require greater myocardial oxygen consumption, leading to myocardial ischemia in the presence of obstructing coronary artery disease. A faster heart rate shortens left ventricular filling times, which may increase left atrial filling pressures, leading to pulmonary edema in patients with systolic or diastolic left ventricular failure. Resting heart rate is a predictor of all-cause and cardiovascular mortality [[24]], with an increase in mean heart rate of 10 beats/min associated with a 9.8% increase in all-cause mortality among patients with coronary artery disease [[25]]. In middle-aged subjects without clinical heart disease, increased night-time heart rate is predictive of increased mortality and cardiovascular risk after adjustment for other cardiovascular risk factors [[26]]. Our previous studies in healthy subjects [[27]-[29]] suggest that UFP inhalation subtly alters both systemic and pulmonary vascular function. Such effects in people with critical cardiac disease could contribute to the observed associations between PM exposure and cardiovascular morbidity and mortality.

Most of the ECG measures in this study did not differ significantly between air and UFP exposure, including time-domain indicators of HRV (rMSSD, SDNN, pNN50), cardiac repolarization (QTc, T wave amplitude), ischemia (ST segment), or arrhythmia. The autonomic dysfunction associated with diabetes may have reduced the ability of these subjects to respond with further reductions in HRV. Also, subjects with known cardiovascular disease or coronary artery disease were excluded.

Clinical exposure studies have shown variable effects of PM on ECG parameters. Some epidemiology and panel studies have shown small effects of PM on heart rate [[30]-[34]], but most have not. We previously studied ECG changes after exposure of healthy subjects to laboratory generated ultrafine carbon particles and clean air [[35]]. We found no effects on heart rate, and generally non-significant effects on HRV, cardiac repolarization, and the ST segment, with trends suggesting increased parasympathetic tone with UFP exposure. Samet et al. [[36]] studied 19 young, healthy subjects exposed to concentrated ambient ultrafine particles and clean air, with intermittent exercise. They found increased frequency-domain markers of HRV, indicating elevated, rather than reduced, vagal (parasympathetic) input to the heart, consistent with our studies with laboratory generated particles in healthy subjects. In contrast, Gong et al. [[37]] found no convincing effects of concentrated ambient UFP, with intermittent exercise, on heart rate or HRV in healthy and asthmatic subjects. Controlled clinical studies in young, healthy subjects of concentrated ambient fine particles, and diesel exhaust [[38],[39]], have shown no consistent effects on HRV. However, studies in elderly subjects have shown reductions in HRV in response to concentrated ambient fine particles [[40]]. Most clinical studies have not looked beyond 24 hours after exposure.

The bulk of evidence that PM exposure reduces HRV comes from panel studies of exposure to ambient air pollution, involving older subjects and those with heart disease. For example, Henneberger and colleagues [[41]] found air pollution effects on cardiac repolarization duration, morphology, and variability in 56 male patients with ischemic heart disease. Pope et al. [[42]] found reductions in HRV associated with increases in concentrations of particulate matter less than or equal to 2.5 μm (PM_2.5_) in elderly subjects. Rich et al. [[43]] studied 76 patients in a cardiac rehabilitation program who had a recent myocardial infarction or unstable angina. Exposures to fine particles and UFP were associated with decreases in parasympathetic modulation, prolongation of late repolarization duration, increased blood pressure, and systemic inflammation. In 28 elderly subjects in Boston, ambient PM_2.5_ exposure was associated with decreased rMSSD and pNN50 [[44]]. There were stronger effects of black carbon on SDNN in subjects with previous myocardial infarction. Associations with heart rate were not reported. Exposures to ambient air pollution while riding in a car reduced HF HRV in 21 people with type 2 diabetes [[45]], although associations of changes in HF HRV with individual pollutant concentrations in the vehicle were not significant.

To the best of our knowledge, this is the first clinical study of the cardiac effects of exposure to UFP in people with diabetes. The strengths of our study include use of a potentially susceptible, under-studied population; the double-blind, randomized, crossover design; housing of the subjects in a controlled environment overnight prior to exposure; and controlled exposures to well-characterized elemental carbon UFP as a surrogate for ambient UFP. In addition, continuous ECG recordings for 48 hours allowed assessment of delayed effects. The limitations include those inherent in human clinical studies [[46]], with a relatively limited number of study subjects, short term exposures, and possible confounding effects of ambient pollutant exposures prior to the experimental exposures. Exposures were performed at rest, and effects may differ with exercise. In addition, our study utilized laboratory generated pure elemental carbon UFP, which differ from ambient UFP. Our elemental carbon particles may underestimate the potential effects of ambient UFP, which represent a complex mixture of chemical species, including organics. However, we feel that studies such as ours using surrogates for ambient UFP have a role in helping to understand UFP health effects. Ultrafine particle concentrators do not efficiently concentrate the smallest of the ambient UFP size fraction, and probably cause alterations in surface chemistry of the particles that do get concentrated. Soluble particles do not get concentrated, and volatile surface components are lost. Thus, concentrated particles must also be considered imperfect surrogates of ambient UFP. The other alternative is studying people undergoing real-world exposures, as in panel studies. Here the exposures involve complex mixtures of pollutants that include larger particles and gases that vary continuously in concentration and composition, and it is impossible to completely sort out the role of UFP in causing any observed effects. Understanding of health effects of UFP exposure requires synthesis of data from various approaches, including clinical studies of laboratory generated and concentrated ambient UFP, panel studies of ambient exposures, epidemiology, and animal exposure studies.

While exposure mass concentrations of ~50 μg/m^3^ used in this study were relevant to ambient PM exposures, the exposure number concentrations were substantially higher than those in most ambient settings. Nevertheless, UFP number concentrations on busy highways can reach peaks within an order of magnitude of those used in this study [[47]].

## Conclusions

Inhalation of elemental carbon ultrafine particles alters heart rate and heart rate variability in people with type 2 diabetes, and suggests that effects may occur and persist many hours after the end of the exposure. These findings are consistent with epidemiology studies showing increased risk of PM exposure in people with diabetes, and provide a potential mechanism for adverse cardiovascular effects.

## Methods

### Study population

The study was approved by Research Subjects Review Board of the University of Rochester Medical Center, and by the US Environmental Protection Agency Human Subjects Research Review Official. Informed, written consent was obtained from all subjects. We recruited 19 subjects who were never-smokers, 30–60 years of age, with type 2 diabetes, as defined by the World Health Organization [[48]]. We attempted to balance the subjects by gender and age (30–45 vs. 46–60 years). Subjects were required to have stable diabetes with no changes in medications for at least three months prior to entry, and they continued on the same regimen during the study. Exclusion criteria included history of clinical cardiovascular disease, cardiac arrhythmias, major organ dysfunction, uncontrolled hypertension, frequent hypoglycemia, statin-type lipid lowering medications, use of β-adrenergic blocker medications, or a history of occupational exposure to particles (e.g. welding, mining, foundry work). Subjects were asked to avoid non-steroidal anti-inflammatory drugs and phosphodiesterase enzyme inhibitors during the study.

### Protocol

The exposure protocol has been previously described [[16]]. This was a double-blind, randomized, crossover study design. Subjects were admitted to the University of Rochester Clinical Research Center (CRC) the day prior to exposure and stayed overnight, to reduce confounding from exposure to outdoor air pollution. The following morning, a 12-lead ECG monitoring device was affixed (see below), blood pressure and heart rate were measured in the seated position using an automated device, and then the subject inhaled either filtered air (0–10 particles/cm^3^) or elemental carbon UFP (~10^7^ particles/cm^3^, ~50 ug/m^3^, count median diameter 32 nm) by mouthpiece, for 2 hours at rest. Blood pressure was measured again 30 minutes and 3.5 hours after exposure. Subjects left the CRC approximately 6 hours after exposure, with instructions to avoid vigorous exercise or polluted environments between visits. They returned approximately 24 and 48 hours after exposure for followup testing, including blood pressure. The alternate exposure occurred at least 3 weeks later, and the order of exposure was randomized.

Our facility and particle inhalation system have been described previously [[49]]. The particles were generated in argon using an electric spark discharge between graphite electrodes, in a modified commercial generator (Palas Co., Karlsruhe, Germany). This produced particles consisting of >95% elemental carbon, free of metals and organic carbon. Particle number (condensation particle counters, model 3220a; TSI, Inc., St. Paul MN), mass (tapered element oscillating microbalance, Rupprecht and Patachnick, Albany, NY), and size distributions (Scanning Mobility Particle Sizer, model 3071; TSI, Inc.) were monitored.

### Continuous ECG

A continuous digital 12-lead ECG was recorded (Mortara Instruments, Milwaukee, MN), beginning approximately 1 hour prior to exposure, for a total of 48 hours. Data were recorded on three different cards (0–9 hours, 9–24 hours and 24–48 hours). The first data card (0–9 hours) included the exposure and approximately 6 hours after exposure. 5-minute ECG analyses were used for detailed HRV analyses, prior to, immediately after, 3.5 hours after, during the night after and 21 and 45 hours after exposure. The daytime 5-minute analysis segments were initiated after the subject was supine, but not sleeping, for 5 minutes, in a quiet room in the Clinical Research Center. These rest periods for the detailed analysis segments preceded other study procedures.

### ECG analyses

Analysis of the 24-hour ECG recordings was performed using a research version of Mortara’s MISHA software, yielding several ECG parameters, including beat-to-beat RR intervals, lead-specific and beat-to-beat ST segment levels, T-wave amplitude, and T-wave complexity. Next, an 8-beat-segment average was computed for the QT interval, adjusted for heart rate with Bazett’s formula [[50]]. T-wave complexity, describing morphology of the T-wave, was measured in each beat by principal component analysis (PCA) based on eight original leads, and averaged over the 5-minute period [[51]]. Variability of T-wave complexity was measured as a standard deviation over the 5-minute period. ST segment analysis focused on leads II, V2, and V5, using the median ST segment level over the 5-minute period.

The time-domain HRV parameters SDNN and rMSSD were calculated for each 5-minute segment of interest, and for a 16-hour period starting 3.5 hours after exposure. The following frequency-domain HRV parameters were computed for each 5-minute segment using a fast Fourier technique: HF and LF, both expressed in normalized units, and the LF/HF ratio [[52]]. We also measured heart rate turbulence (HRT) and deceleration capacity (DC) across the whole session, as previously described [[43]]. Not all recordings had at least 1 premature ventricular beat (mean 126 ventricular ectopic beats per recording), so HRT analyses were done on a subset of recordings. Otherwise, all other outcomes were measured in all recordings.

### Statistics

This study utilized a randomized, two-by-two crossover design in which each subject was exposed to both particles and air. The study was balanced for gender. A washout period of at least 3 weeks between the exposures was included to eliminate carry-over effects.

The statistical method used to analyze the endpoints in this study was based on the method of Jones and Kenward [[53]] for the analysis of cross-over trials with repeated measurements within treatment (exposure) periods. There were two types of covariance patterns among measurements from the same subject: correlations among measurements in the same treatment period at different time points, and correlations among measurements from different treatment periods. We reasonably assumed that the between- and within-period covariance structures are separable. To model these two covariance structures, a linear mixed model was used in which random subject effect accounted for the between-period dependencies and an autoregressive lag 1 correlation structure was used for the within-period dependencies.

Adjustments for the baseline measurements taken at the start of each session were made by including the baseline measurement as a term in the model for the mean response.

We ran two models: The first model examined the primary hypothesis of treatment (exposure) effects and contained terms for treatment, period, time, and time-by-treatment interactions. The second explored whether the exposure effects differed based on gender and age (≤45 years vs. > 45 years), and contained terms for treatment, gender, age group, treatment-by-gender interactions, and treatment-by-age interactions.

## Competing interests

The authors declare that they have no competing interests.

## Authors’ contributions

RV prepared the first draft of the manuscript and performed arterial blood sampling. WZ analyzed the ECG recordings and assisted in data interpretation and manuscript review. MU assisted in study design, subject supervision, data interpretation, and manuscript review. AP assisted with subject supervision and arterial blood sampling. DC supervised the exposure chamber operation and conducted the randomization. EL was the study coordinator and assisted in data analysis and manuscript preparation. DO, JB, and JW performed the biostatistical analyses. MF was the principal investigator, assisted in manuscript preparation, assisted with subject supervision, and performed arterial blood sampling. All authors read and approved the final manuscript.
